# Reviewer acknowledgement 2015

**DOI:** 10.1186/s40246-016-0064-4

**Published:** 2016-02-15

**Authors:** Vasilis Vasiliou

**Affiliations:** Department of Environmental Health Sciences, Yale School of Public Health 60 College Street, New Haven, CT 208034 USA

## Abstract

The Editors of *Human Genomics* would like to thank all our reviewers who have contributed to the journal in volume 9 (2015).

**Luisa Azevedo**

Portugal

**Chad Brocker**

United States of America

**Georgia Charkoftaki**

United States of America

**Zissis Chroneos**

United States of America

**Stephen Curry**

United States of America

**Daniela Del Gaudio**

United States of America

**Andrew Dewan**

United States of America

**Lynnette Robin Ferguson**

New Zealand

**Kristofer Fritz**

United States of America

**Christos Hatzis**

United States of America

**Xiu Huang**

United States of America

**Paraskevas Iatropoulos**

Italy

**Chee Seng Ku**

Singapore

**Poh San Lai**

Singapore

**Tukiet Lam**

United States of America

**Jun Li**

United States of America

**Beata Lipska**

Poland

**Xiaochuan Liu**

United States of America

**Jirong Long**

United States of America

**Qianyi Ma**

United States of America

**Brian Meyer**

Saudi Arabia

**Andrew Monte**

United States of America

**Daniel Nebert**

United States of America

**Michael Nothnagel**

Germany

**A. Bilge Ozel**

United States of America

**Eric Pasmant**

France

**George Patrinos**

Greece

**Tzu Lip Phang**

United States of America

**Michele Ramsay**

South Africa

**Juergen Reichardt**

Ecuador

**Daniela Saggioro**

Italy

**Robert Scheinman**

United States of America

**Jadranka Sertic**

Croatia

**David Sinclair**

United States of America

**Surendra Singh**

United States of America

**Sumio Sugano**

Japan

**Peter Taschner**

Netherlands

**Eleni Tomazou**

Austria

**Jeffrey Townsend**

United States of America

**Panagiotis A. Tsonis**

United States of America

**Argyrios Tzouvelekis**

United States of America

**Nong Van Hai**

Vietnam

**Athanassios Vasilopoulos**

United States of America

**Alkis Vatopoulos**

Greece

**Carsten Wiuf**

Denmark

**Tongbin Wu**

United States of America

**Zheng Xia**

United States of America

**Fan Xiucheng**

United States of America

**Shuhua Xu**

China

**Xiaoqing Yu**

United States of America

**Yawei Zhang**

United States of America

**Guojie Zhang**

China

**Hongyu Zhao**

United States of America

**Yong Zhu**

United States of America

